# Melatonin Modifies Histone Acetylation during *In Vitro* Maturation of Mouse Oocytes 

**DOI:** 10.22074/cellj.2018.4860

**Published:** 2018-03-18

**Authors:** Somayeh Keshavarzi, Mohammad Salehi, Fattaneh Farifteh-Nobijari, Taher Hosseini, Sara Hosseini, Alaleh Ghazifard, Marefat Ghaffari Novin, Vahid Fallah-Omrani, Mohsen Nourozian, Ahmad Hosseini

**Affiliations:** 1 Cellular and Molecular Biology Research Center, Shahid Beheshti University of Medical Sciences, Tehran, Iran; 2 Department of Biology and Anatomical Sciences, Faculty of Medicine, Shahid Beheshti University of Medical Sciences, Tehran, Iran; 3 Department of Biotechnology, Faculty of Medicine, Shahid Beheshti University of Medical Sciences, Tehran, Iran; 4 Department of Biotechnology, School of Advanced Technologies in Medicine, Shahid Beheshti University of Medical Sciences, Tehran, Iran

**Keywords:** Glutathione, In Vitro Oocyte Maturation, Melatonin, Reactive Oxygen Species

## Abstract

**Objective:**

We evaluated the effect of melatonin, as a potent antioxidant agent, on glutathione (GSH) and reactive oxygen species (ROS) levels, as well as histone H3 lysine 9 (H3K9), and H4 lysine 12 (H4K12) acetylation when added to oocytes culture medium.

**Materials and Methods:**

In this experimental study, two *in vitro* and *in vivo* groups were used. In the *in vitro* group, cumulus oocyte complexes (COCs) from the ovaries of B6D2F1 mice were cultured in maturation medium containing two doses of melatonin (10^-9^ and 10^-6^ M) and without melatonin [control group treated with dimethyl sulfoxide (DMSO)] for 22-24 hour. The cumulus expansion and nuclear status were monitored by an inverted microscope. Next, COCs were isolated from the oviducts of superovulated mice and studied as the *in vivo* group. In *in vitro* and *in vivo* matured oocytes, GSH and ROS levels were assessed by monochlorobimane (MCB) and 2-7-dichlorodihydrofluorescein diacetate (H2DCFDA) staining, respectively. Changes in histone acetylation were examined by immunofluorescent staining with specific antibodies against acetylated H3K9 and H4K12.

**Results:**

The H4K12 acetylation and ROS levels were significantly higher in the oocytes matured in the in vitro group compared to the *in vivo* group (P<0.05). Furthermore, glutathione levels in the *in vitro* group were considerably lower than that of the *in vivo* group (P<0.05). Melatonin at the concentration of 10^-6^ M had the most substantial effect on nuclear maturation and histone acetylation as well as glutathione and ROS levels in the *in vitro* group (P<0.05).

**Conclusion:**

Exogenous melatonin improves the competence of mouse oocytes during *in vitro* maturation (IVM).

## Introduction

*In vitro* maturation (IVM) is a protocol that minimizes the amount of drugs used in patients and prohibits ovarian hyper-stimulation. It is applied in lieu of conventional *in vitro* fertilization (IVF). In women who are undergoing chemotherapy, IVM could be recommended for preservation of fertility by cryopreservation of reproductive germ cells. Moreover, poor responders can benefit from this treatment as they do not require receiving high doses of gonadotropins (1). However, the success rate of the IVM method is low (2).

It is not clear whether oocytes matured *in vitro* are intrinsically damaged or if culture conditions are incapable of supporting complete developmental potential of the oocytes (3). “Oocyte maturation is one of the most critical periods for normal development and differentiation for an individual” (4). Composition of the culture medium is one of the important factors affecting the nuclear maturation, cleavage, and blastocyst formation (5).

Environmental factors (e.g. exposure to light and increased oxygen concentration) can cause oxidative stress in embryo and oocyte (6). Reactive oxygen species (ROS) may induce damage to cell membranes, and DNA, and can also cause apoptosis (7). It has been proven that when the time after ovulation increases, higher levels of ROS accumulate in the oocytes, both *in vitro* and *in vivo* (8). Glutathione (γ-glutamylcysteinylglycine, GSH), a significant intracellular free thiol with an antioxidant property, protects the cells against oxidation, and by increasing the time post-ovulation, GSHs are reduced, in vitro (9). 

Changes in the antioxidant capacity of cells by metabolic oxidants (alterations in the level of GSH production) influence their development (10). Allen and Balin (10) suggested that free radicals can change gene expression. Gene expression can be dynamically controlled by epigenetic processes without alteration of DNA sequences (11). Epigenetic reprogramming occurs in the periods of gametogenesis and embryogenesis and includes methylation, histone acetylation, phosphorylation, and poly ubiquitination (12). 

Histone acetylation is a dynamic process occurring in histone H3 and H4 during mammalian oocyte maturation and is known to play vital roles in different cellular functions (13). Histone modifications are enzymatic processes catalyzed by two enzymes, histone acetyltransferases (HATs) and histone deacetylases (HDACs) (14). In mouse oocytes, histones H3 and H4 are completely deacetylated at MII by HDAC activity (15). Prevention of histone deacetylation can cause aneuploidy in fertilized mouse oocytes, resulting in the death of embryos in utero at an early stage of development (16). Factors such as oxygen and ROS as well as changes in GSH levels, alter the chromatin structure (10).

Antioxidants, by scavenging free radicals in *in vitro* culture medium, regulate the levels of free radicals and improve culture conditions (17). Melatonin (N-acetyl5- methoxytryptamine), an indole amine with a strong antioxidant property, is secreted by the pineal gland into the oviduct and follicular fluid during ovulation (18). In the maturation medium, melatonin, by reducing ROS, induces oocyte maturation and blastocyst formation (19, 20). Beside directly scavenging ROS, melatonin adjusts the expression of antioxidant enzymes and genes by epigenetic mechanisms (21). Therefore, the aim of this study was to investigate the following factors during oocyte maturation: First, changes in ROS production and its role in altering GSH levels were investigate; Second, the effects of ROS and GSH on epigenetic alterations were studied; and Third, the effective dose of melatonin in reducing ROS, glutathione production, and epigenetic changes were calculated.

## Materials and Methods

All animal experiments were performed in accordance with the Animal Experiment Standards adopted by the Shahid Beheshti University of Medical Sciences committee on bioethics (16-94/12/2). Except otherwise noted, all chemicals and reagents used in this research were purchased from Sigma Chemical Corporation (St. Louis, MO, USA).

### Oocyte collection 

In this experimental study, for the *in vitro* group, the oocytes were obtained from female mice B6D2F1 aged between 6 and 8 weeks old and purchased from Pasteur Institute, Tehran, Iran. Animals were kept under controlled light/dark and temperature conditions with free access to water and food. They received 10 IU of pregnant mare serum gonadotropin (PMSG). Forty-eight hours later, mice ovaries were excised, and cumulus oocyte complexes (COCs) were collected by aspiration of ovaries, using a 28-gauge needle, in tissue culture medium (TCM)-199-HEPES supplemented with 5% foetal bovine serum (FBS). For the *in vivo* group, the mice were super-ovulated as described previously (22). Briefly, the female mice were intraperitoneally injected with human chorionic gonadotrophin (hCG, Pregnyl®, Organon) 48 hours after PMSG injection. After 12-14 hours, the mice were killed, and the COCs were isolated from the oviducts and put into HTCM medium, which contained 100 IU/ml hyaluronidase, to separate cumulus cells from the oocytes. The oocytes were applied in the following experiments.

### *In vitro* maturation

The COCs were washed three times in maturation medium containing TCM-199 supplemented with 10% FBS, 0.2 mM sodium pyruvate, 2 mM L-glutamine, 10 µg/mL follicle stimulating hormone (FSH), 10 µg/mL luteinizing hormone (LH), and 1 µg/mL 17ß- estradiol.

Oocyte maturation was performed by culturing approximately 10-15 oocytes for 22-24 hours in 50 µl maturation medium droplet under mineral oil at 37°C, in a humidified atmosphere with 5% CO_2_ as previously described (23, 24). Melatonin stock solution was prepared using an ethanol/TCM-199 system with serial concentrations. Thus, 10^-6^ and 10^-9^ M melatonin solutions were prepared. dimethyl sulfoxide (DMSO) was used at the same concentration as 10^-6^ M melatonin.

### Assessment of the cumulus expansion and nuclear maturation 

The expansion of COCs were assessed by inverted microscope (Olympus, Japan) 22-24 hours of starting IVM. Oocytes were denuded in hyaloronidase and then classified as germinal vesicle (GV), metaphase I (MI), and metaphase II (MII). The oocytes with polar body were considered matured oocytes (metaphase-II).

### Measurement of reactive oxygen species and glutathione levels in oocytes

MII stage oocytes were sampled from *in vitro* and *in vivo* groups to determine their intracellular ROS. Briefly, 2-7-dichlorodihydrofluorescein diacetate (H2DCFDA, Invitrogen, USA) was used to evaluate intracellular ROS levels according to the intensity of green fluorescence (25). For each group, a total of 30-35 oocytes were incubated for 30 minutes in the dark with phosphate-buffered saline (PBS) which contained 1 mg/ml polyvinyl alcohol (PBS/ PVA) containing 10 µM H2DCFDA.

After incubation, the oocytes were washed with PBS/ PVA and placed in 10-µl droplets; then, the fluorescence was observed, using fluorescence microscope equipped with UV filters (460 nm). The recorded fluorescent images were analyzed by ImageJ software 1.33 u (National Institutes of Health, Bethesda, MD, USA). Monochlorobimane (MCB) was employed to detect the GSH levels in oocytes according to the intensity of blue fluorescence (26). The matured oocytes of *in vitro* and *in vivo* groups were incubated with flushing holding medium (FHM) supplemented with 50 mM MCB for 45 minutes, washed with PBS-PVA, and placed into a 10-µl droplet. Fluorescence was observed through a fluorescence microscope equipped with a UV filter (390 nm). The fluorescence intensity was measured as described above by ImageJ software. 

### Immunocytochemistry

Immunofluorescent staining was conducted according to a previously published study (27). The experiment was performed three times. Here, 20 oocytes at MII stage, were washed in PBS containing 3 mg/ml PVP (PBS/ PVP), fixed for 1 hour in 4% paraformaldehyde in PBS, and permeabilized by incubation with 0.2% Triton X-100 in PBS for 30 minutes at room temperature. The cells were blocked in PBS/5% bovine serum albumin (BSA) for 45 minutes and incubated with primary antibody against antiacetyl H4/K12 (H4K12ac, Abcam, UK) (1:200 dilution) and H3/K9 (H3K9ac, Abcam, UK) (1:500 dilution) at 4°C overnight. The following day, after washing three times with PBS/PVA for 5 minutes, the oocytes were incubated with the secondary antibody conjugated with FITC for 1 hour at 37°C. Then, DNA was incubated with 3 mg/ml 4, 6-diamidino-2- phenylindole (DAPI) for 20 minutes, and the cells were mounted on glass slides with etched rings to prevent them from being ruptured by the cover slip. The slides were imaged using a fluorescent microscope. Fluorescence intensity was determined by ImageJ software. 

### Statistical analysis

All statistical analyses were performed using Service Provisioning System Software (SPSS) 22 for windows (SPSS, Chicago, IL, USA). The means of MII, cumulus expansion, and MI were compared by non-parametric analysis test (Kruskal-Wallis). Acetylation of histones as well as glutathione and ROS levels in both *in vitro* and *in vivo* groups were compared by Analysis of Variance (ANOVA). The data were expressed as means ± SD. A P<0.05 was considered significant.

## Results

### Oocyte maturation

818 COCs were cultured in TCM medium containing two concentrations of melatonin (10^-6^ and 10^-9^ M) and without melatonin (control and DMSO) for 22-24 hours. We observed that the number of metaphase II oocytes was significantly higher in medium supplemented with melatonin 10-6 M compared to the control group and the other groups (85 and 64%, respectively). However, in TCM medium supplemented with melatonin 10^-9^ M, the rate of nuclear maturation significantly decreased compared to the control group (37%). The lowest degree of cumulus expansion was noticed in the group treated with melatonin 10^-9^ M (41%) which was significant different from those of the control (83%) and melatonin 10^-6^ M groups (87%). The rate of metaphase I arrest was significantly higher in the oocytes treated with melatonin 10^-9^ M compared to the control and the other groups (62 and 35%, respectively, Table 1).

### Reactive oxygen species and glutathione levels in oocytes during *in vivo* and *in vitro* maturation

**Fig.1:**
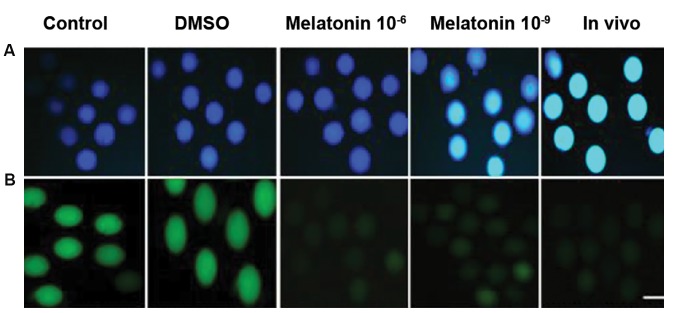
Florescence intensity of oocytes in different groups (*in vitro* and *in vivo*). A. Stained with monochlorobimane (MCB) to determine the level of intracellular glutathione (GSH) and B. 2-7- dichlorodihydrofluorescein diacetate (H2DCFDA) to detect reactive oxygen species (ROS) (bar=50 µm). DMSO; Dimethyl sulfoxide.

**Fig.2:**
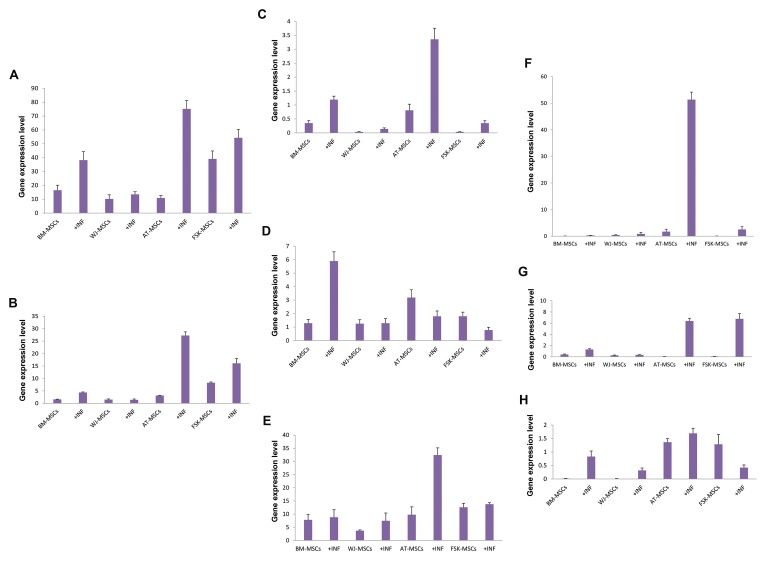
Glutathione level in matured oocytes in *in vitro* (control, DMSO, melatonin 10^-6^ M and melatonin 10^-9^ M) and *in vivo* groups. The bar with different letters were significantly different (P<0.05). DMSO; Dimethyl sulfoxide.

The intracellular GSH level of mouse oocytes was evaluated in different groups (50 oocytes in each) (Fig .1A). The results indicated that the GSH level was significantly higher in the *in vivo* group compared to in vitro groups (P<0.05, Fig .2). However, the GSH level in the group treated with melatonin 10^-6^ was significantly improved (199.26 ± 9.13) compared to the other *in vitro* groups (P<0.05).

**Fig.3:**
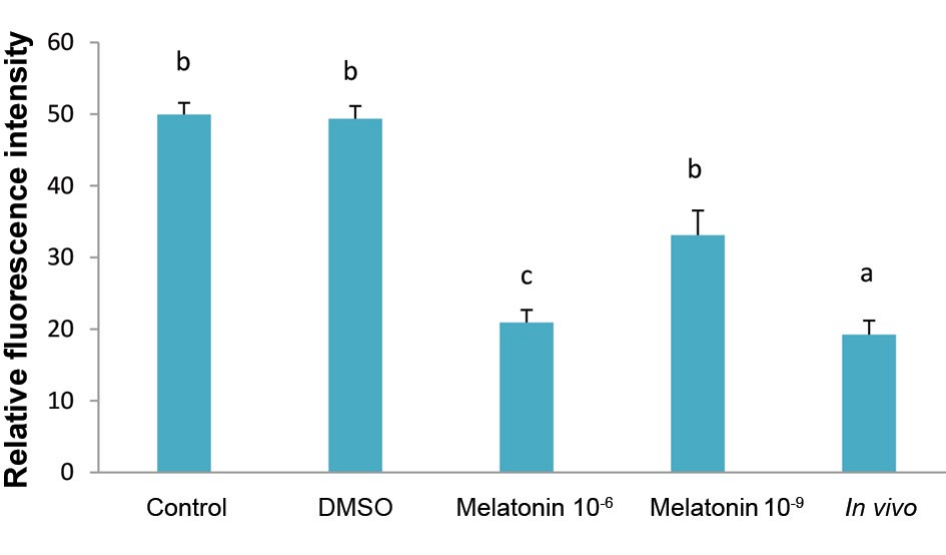
ROS level in matured oocytes in *in vivo* and *in vitro* (control, DMSO, melatonin 10^-6^ M and melatonin 10^-9^ M) groups. The bar with different letters were significantly different (P<0.05). ROS; Reactive oxygen species and DMSO; Dimethyl sulfoxide.

To determine the ROS levels in oocytes (30 oocytes in each group), the H2DCFDA staining was carried out (Fig .1B). The ROS level was significantly lower in the in vivo group compared to *in vitro* groups. The results indicated that the ROS level in the oocytes significantly decreased in the group treated with melatonin 10^-6^ M compared to *in vitro* groups (P<0.05, Fig .3).

**Table 1:** Effect of melatonin on cumulus expansion and nuclear maturation in mouse oocytes


Group	Number of COCs	MI n (mean ± SD)	Cumulus expansionn (mean ± SD)	MIIn (mean ± SD)

Control	198	70 (35.35 ± 2.62)^a,b^	165 (83.33 ± 6.39)^d^	128 (64.65 ± 4.87)^f, g^
DMSO	205	95 (43.34 ± 3.20)	137 (66.83 ± 4.34)	110 (53.66 ± 2.82)
Melatonin 10^-6^ M	222	32 (14.41 ± 2.14)^a, c^	194 (87.39 ± 5.56)^e^	190 (85.59 ± 5.96)^f, h^
Melatonin 10^-9 ^M	193	121 (62.69 ± 3.12)^b,c^	81 (41.97 ± 2.07)^d, e^	72 (37.31 ± 2.00)^g, h^


Within the same column, values with the same letters were significantly different (P<0.05). DMSO; Dimethyl sulfoxide, COCs; Cumulus-oocyte complex, MI; Metaphase I, and MII; Metaphase II.

### Histone acetylation during *in vitro* and *in vivo* maturation of MII oocytes

**Fig.4:**
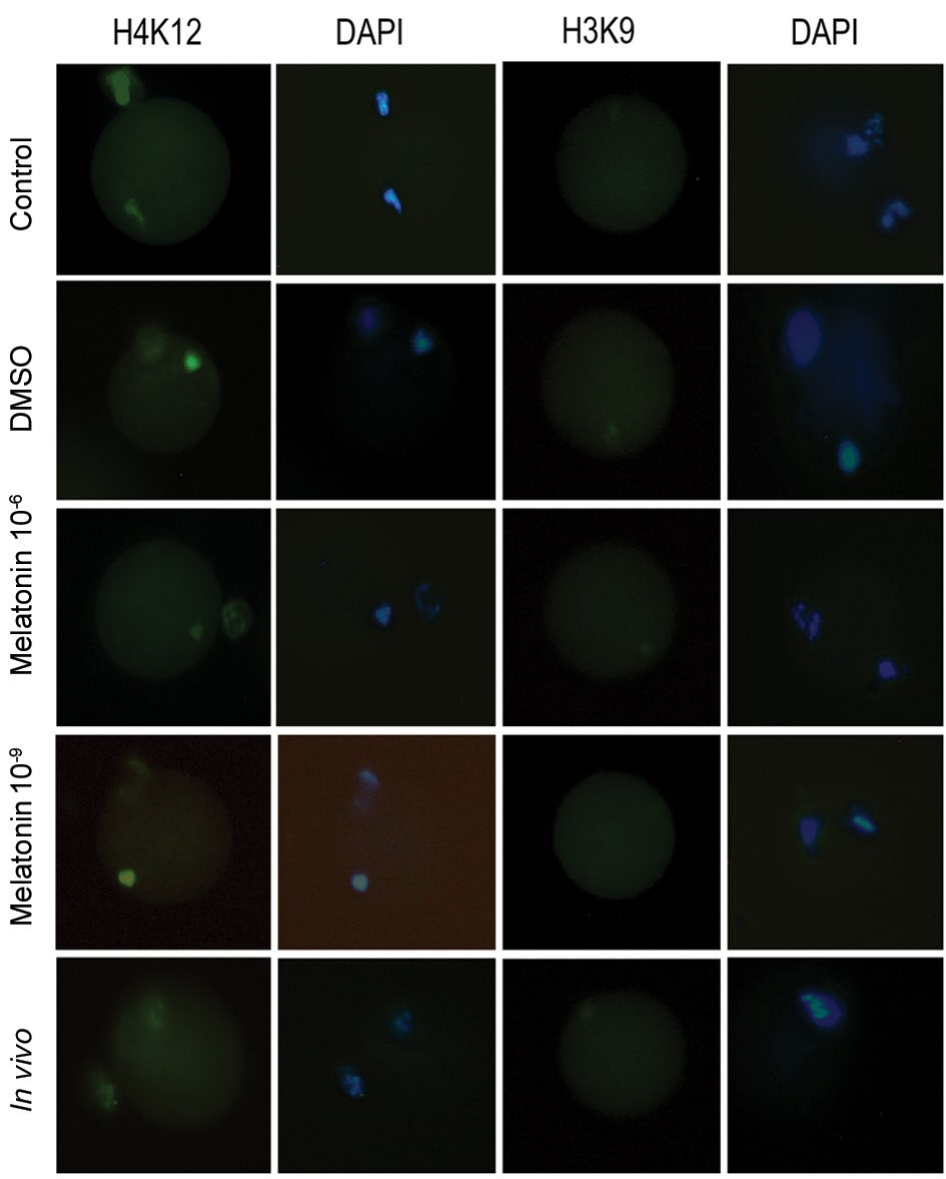
Immunofluorescence staining for H4K12ac (green) and H3K9ac (no signal) in MII oocytes of *in vitro* (control, DMSO, melatonin 10^-6^ M, melatonin 10^-9^ M) and *in vivo* groups. DNA was stained with DAPI (blue) (bar=20 µm). DMSO; Dimethyl sulfoxide.

The acetylation of H3K9 and H4K12 were evaluated based on the immunofluorescence intensity in different groups (Fig .4). 

**Fig.5:**
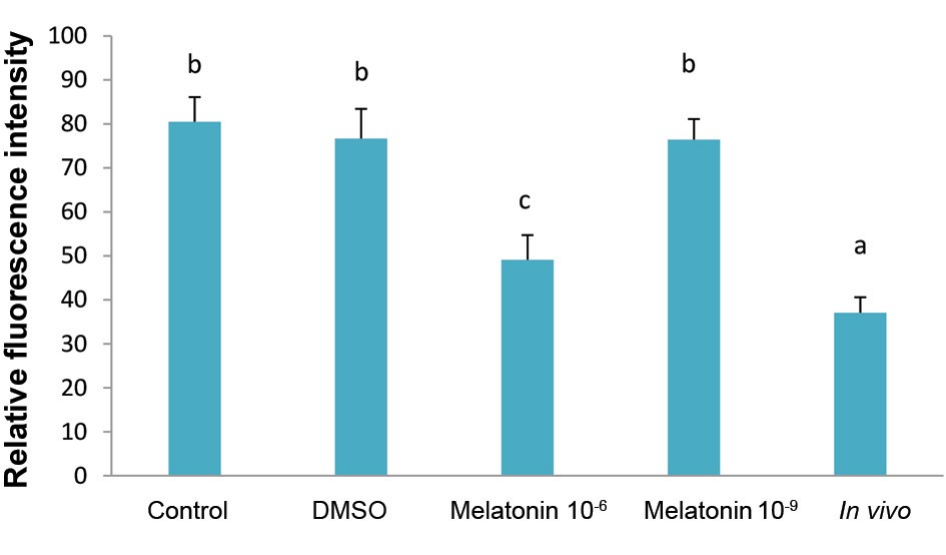
Acetylation level of H4k12 in matured oocytes of *in vivo* and *in vitro* (control, DMSO, melatonin 10^-6^ M, melatonin 10^-9^ M) groups. The bar with different letters were significantly different (P<0.05). DMSO; Dimethyl sulfoxide.

*In vitro* maturation of oocytes significantly increased the acetylation level of H4K12 compared to *in vivo* oocytes (P<0.05, Fig .5). However, treatment with melatonin 10^-6^ M significantly reduced the H4K12 acetylation compared to the other *in vitro* groups (P<0.05, Fig .5). No fluorescence signals for AcH3K9 were detected during in vitro and *in vivo* maturations (Fig .4).

## Discussion

In the present study, we demonstrated useful effects of melatonin on the oocyte’s markers of quality and oxidative stress, and showed evidence of H4K12ac alterations in oocytes during *in vitro* maturation. The process of oocyte maturation has two steps including nuclear and cytoplasmic maturations. Nuclear maturation was visualized by the extrusion of the second polar body. However, ‘’the cytoplasmic maturation involved organelle reorganization, activation of metabolic pathways, storage of mRNAs, proteins, and transcription factors for normal fertilization, cell cycle progression, and activation of genetic and epigenetic programs of preimplantation embryonic development’’ (28).

Under *in vitro* conditions, an imbalance between the formation and removal of oxidants, affects the maturation and cleavage of embryos (6). On the basis of the results of various studies, melatonin (N-acetyl 5-metoxy tryptamine) is potentially beneficial under culture conditions as a direct scavenger of free radicals (29). Melatonin stimulates the activity and expression of a number of antioxidant enzymes and inhibits the activity of pro-oxidant enzymes (30).

We focused on the effects of melatonin and their correlation with the nuclear maturation of oocytes. From this perspective, our results revealed high-rates of polar body extrusion in oocytes treated with melatonin 10^-6^ M and low dose (10^-9^ M) compared against outcome on oocyte maturation shown in previous studies (23). Thus, antioxidants at certain concentration are crucial for oocyte maturation, as consistently shown by other researchers (16). During *in vitro* maturation, intracellular glutathione concentration is an important biomarker to assess the degree of cytoplasm maturation, viability, and developmental capacity in mammalian oocytes (31) as GSH has a pivotal role in maintaining redox homeostasis and scavenging peroxides (32).

We consequently assessed the level of ROS, in relation to GSH level under *in vitro* conditions; we observed that vitro conditions were associated with increased production of ROS, and decreased levels of intracytoplasmic GSH in oocytes during *in vitro* maturation in comparison to in vivo maturation. Probably, this occurs because oocytes during in vitro maturation, in comparison to oocytes during *in vivo* maturation, are exposed to higher oxygen concentrations. This is because oxygen concentration during *in vitro* maturation is three-times higher compared to the lumen of the female reproductive tract. Higher concentration of O_2_in in vitro cultures, compared to *in vivo* cultures, produces higher levels of ROS such as superoxide and hydrogen peroxide (H_2_O_2_) (33). Melatonin plays a critical role in protecting the oocytes against oxidative stress through its radical-scavenging activity, resulting in enhancement of cytoplasmic maturation (18, 20).

Intracytoplasmic decrease of ROS as well as elevated GSH levels of oocytes in the group treated with melatonin 10^-6^ M would explain a higher quality and rate of maturation of oocytes. Thus, we hypothesized that melatonin at higher concentrations may have a more marked radical-scavenging activity, resulting in lower levels of ROS, and higher GSH level. 

Oxygen and glutathione could effectively regulate and protect the epigenotype of cells. Altering the level or synthesis of GSH in cells by impacting both DNA and histone methylation influences the epigenotype of cells (11). By changing chromatin structure, oxygen and ROS also influence gene expression (10). In previous studies, the effect of GSH level on DNAmethylation was surveyed (34). In this study, we assumed that a similar effect would be observed for histone acetylation. Recent studies also suggest that IVF and IVM may have negative impacts the epigenetic status in oocytes (35). Thus, maturation of oocytes is sensitive to environmental changes that can cause epigenetic alterations, deregulation of gene expression, and finally, embryo defects or loss (4).

The acetylation level of H4K12 in porcine oocytes is significantly increased by superoxide overproduction (36). In addition, Huang et al. (37) showed that the acetylation level of H4k12 in mouse oocytes increases during *in vivo* and *in vitro* postovulatory aging, but acetylation was not found at lysine 9 in H3. Our immunocytochemical analysis of histone acetylation identified a strong signal of H4K12 in oocytes during *in vitro* maturation when compared to in vivo matured oocytes, which accords with the changing patterns of ROS content and GSH level in oocytes. However, acetylation, similar to *in vivo* maturation, was not found in H3K9 during *in vitro* maturation.

Two explanations could account; first, the expression of HDAC1 mRNA in MII oocytes decreases during in vitro maturation, but does not disperse (4) and Second, other acetyltransferases or deacetylases might complete the function of HDAC1 in the regulation of histone modification (38). Furthermore, our study, for the first time, revealed that the level of H4K12 acetylation decreases in oocytes treated with melatonin 10^-6^ M in maturation medium. Melatonin is a highly lipophilic molecule that easily passes through cell membranes and reaches the nucleus. Melatonin might pile up in the nucleus, and interact with specific nuclear binding sites (39). Melatonin, by scavenging the reactive oxygen and nitrogen species in cancer cells, inhibits DNA methyltransferase (DNMT) and regulates the epigenetic status (40).

Therefore, we hypothesized that melatonin may also have an effect on histone acetylation of oocytes. Ultimately, our study revealed that although melatonin during IVM improves nuclear maturation and cytoplasmic maturation and decreases the level of histone acetylation by decreasing ROS, there is a significant difference between *in vitro* and *in vivo* groups. Thus, to obtain a better result, it is recommended that beside melatonin, other factors with the capability of improving cytoplasm maturation and decreasing histone acetylation, should be considered. 

## Conclusion

The results of the present study demonstrated that using exogenous melatonin during *in vitro* maturation increases the nuclear and cytoplasmic maturations and decreases the level of H4K12 acetylation; however, the cytoplasmic maturation, ROS and H4K12 acetylation levels in the *in vitro* group were significantly different from those observed in the *in vivo* group.
